# Efficacy and Safety of Intranasal Ketamine for Acute Pain Management in the Emergency Setting: A Systematic Review and Meta-Analysis

**DOI:** 10.3390/jcm10173978

**Published:** 2021-09-02

**Authors:** Yee Sin Seak, Junainah Nor, Tuan Hairulnizam Tuan Kamauzaman, Ariff Arithra, Md Asiful Islam

**Affiliations:** 1 Department of Emergency Medicine, School of Medical Sciences, Universiti Sains Malaysia, Kubang Kerian 16150, Kelantan, Malaysia; yeesin0310@gmail.com (Y.S.S.); hairulnizam@usm.my (T.H.T.K.); arithra@usm.my (A.A.); 2 Hospital Universiti Sains Malaysia, Kubang Kerian 16150, Kelantan, Malaysia; 3 Department of Haematology, School of Medical Sciences, Universiti Sains Malaysia, Kubang Kerian 16150, Kelantan, Malaysia

**Keywords:** acute pain, intranasal, ketamine, analgesia, emergency

## Abstract

Due to overcrowding, personnel shortages, or problematic intravenous (IV) cannulation, acute pain management is often sub-optimal in emergency departments (EDs). The objective of this systematic review and meta-analysis was to evaluate the efficacy and safety of intranasal (IN) ketamine for adult acute pain in the emergency setting. We searched and identified studies up to 21 May 2021 via PubMed, Scopus, Web of Science, Cochrane Database, and Google Scholar. The random-effects model with 95% confidence intervals (CIs) was used to estimate mean differences (MDs) and odds ratios (ORs). The *I*^2^ statistic and Cochran’s Q test were used to determine heterogeneity. The protocol was registered in PROSPERO (CRD42020213391). Seven randomised controlled trials were included with a total of 1760 patients. There was no significant difference in pain scores comparing IN ketamine with IV analgesics or placebo at 5 (MD 0.94, *p* = 0.26), 15 (MD 0.15, *p* = 0.74), 25 (MD 0.24, *p* = 0.62), 30 (MD −0.05, *p* = 0.87), and 60 (MD −0.42, *p* = 0.53) minutes. There was also no significant difference in the need for rescue analgesics between IN ketamine and IV analgesics (OR 1.66, 95% CI: 0.57−4.86, *p* = 0.35, *I*^2^ = 70%). Only mild adverse effects were observed in patients who received IN ketamine. Our results suggest that IN ketamine is non-inferior to IV analgesics and may have a role in acute pain management among adults in the ED.

## 1. Introduction

Acute pain is one of the most frequent presentations to the emergency departments (ED). Between 2000 and 2010, pain was the leading complaint in 45.4% of the total ED visits, and patients reported pain as a symptom twice as high as those recorded by healthcare professionals (40% vs. 20%) [1]. A wide range of pain diagnoses is brought to EDs, including acute injuries (30%), infections (14%), abdominal pain (12%), chest discomfort (12%), and others (16%) [2]. The most common pain management challenge in the ED is oligoanalgesia (underuse of analgesics). Despite the availability of a variety of analgesics, pain is nevertheless undertreated [3]. Inadequate management tends to negatively impact patients’ quality of life, physical and mental functions. Underuse of analgesics in the EDs can lead to worsened pain and frequent hospital visits, resulting in an enormous burden of cost. Patients with severe acute pain may develop chronic pain as a consequence of oligoanalgesia [4].

Emergency staff shortage, limited beds, and inadequate patient monitoring are the critical management issues in overcrowded ED, which necessitate the use of analgesics that are easier to deliver, faster and with fewer adverse effects [5]. In the ED, delayed pain management is common and usually provided after the patient’s clinical assessment. The initial analgesic drug is usually administered after 70 to 90 min of waiting time, and the dosage is often inadequate to relieve pain [6,7]. In addition, difficult intravenous (IV) cannulation delays pain relief in the emergency setting [8].

The opioid abuse and addiction problems are rapidly growing in the United States (U.S.). In 2017, approximately 47,000 people died from opioid overdose in the U.S. [9]. Opioids are the mainstay of analgesic therapy for moderate to severe pain in the emergency setting. However, patients with hemodynamic instability or potential respiratory and airway compromise requires close monitoring when given opioid. Despite its effectiveness, the available data revealed a strong correlation between opioid prescriptions and opioid-related fatalities. This is due to opioid side effects such as dependence, tolerance, respiratory suppression, and hypotension, especially in situations of opioid overdose [10,11]. The American College of Emergency Physicians (ACEP) recommends a multimodal approach to acute pain management that incorporates pharmacologic and nonpharmacologic therapies. Unless contraindicated or non-opioid pain refractory, it should start with non-opioids [12].

Ketamine has been extensively used for procedural sedation and as an induction agent for rapid sequence intubation (RSI) since its introduction to medicine in the 1960s due to its safe hemodynamic profile [13]. The anesthetic and analgesic effects of ketamine are primarily mediated through non-competitive antagonism of the N-methyl-D-aspartate (NMDA) receptor Ca^2+^ channel pore in the central nervous system (CNS) and spinal cord. It also reduces the presynaptic release of excitatory neurotransmitter (glutamate). Furthermore, ketamine and its metabolites have a ten-fold lower affinity for opioid receptors (μ, δ, and κ receptors) than the NMDA receptor and antagonistic activity for nicotinic, muscarinic, and monoaminergic receptors [14].

Ketamine’s effects are dose-dependent, where the analgesic dosage for pain relief without sedation is about 10–30% of the dissociative sedative dosage. The typical ketamine analgesic dose without sedation is 0.1 to 0.3 mg/kg IV, 0.5 mg/kg intramuscular (IM) and 1 mg/kg intranasal (IN) as compared to 1 to 2 mg/kg IV, 3 to 4 mg/kg IM and 6 to 9 mg/kg IN for the ketamine dissociative sedation dose [15,16,17]. IN ketamine has a bioavailability of 25–50%, and the analgesic effects begin within ten minutes of administration and may last up to 60 min [18,19]. The mild and transient side effects of IN ketamine are fatigue, dizziness, euphoria, and nausea, which usually does not require treatment [18]. IN ketamine is a quick and non-invasive method of drug administration and provides an analgesic effect in acute pain. It might be used in overcrowded EDs and prehospital situations where IV cannulation is not required or difficult [20]. Moreover, a systematic review of ketamine’s analgesic effect on burn injuries in adults found its effectiveness in pain relief and reduced secondary hyperalgesia compared to opioids. The combination of ketamine and morphine treatment reduced the windup phenomenon [21].

Ketamine is extensively utilised in adult populations as an adjuvant to opioid pain therapy to reduce opioid use. In addition, ketamine is being studied as a first-line analgesic treatment for acute pain to minimise opioid consumption [22]. Recent randomised controlled trials (RCTs) have evaluated IN ketamine’s analgesic effectiveness and safety in the adult population. Therefore, the objective of this systematic review and meta-analysis (SRMA) was to evaluate the efficacy and safety of IN ketamine in the management of acute pain among adults in the emergency department. We evaluated the efficacy of IN ketamine by assessing the pain score reduction and the requirement for rescue analgesics. In addition, the prevalence of adverse events was analyzed to assess the safety of IN ketamine in acute pain management.

## 2. Methods

We conducted an SRMA to assess the efficacy and safety of IN ketamine as an analgesic agent for acute pain management among adults in ED. This SRMA was conducted in accordance with the protocol registered with PROSPERO (CRD42020213391). The methodology and reporting were constructed based on the updated guideline of the preferred reporting items for systematic reviews and meta-analyses (PRISMA) guideline [23].

### 2.1. Data Sources and Searches

Studies published from inception to 21 May 2021 were searched and identified via electronic databases of PubMed, Scopus, Google Scholar, Web of Science, and Cochrane Library. Table S1 summarises the search strategy. We searched the RCT reference lists for further potential studies to include in the SRMA. In addition, the World Health Organization’s (WHO) International Clinical Studies Registry Platform and ClinicalTrials.gov were used to search for completed and ongoing trials. Duplicate studies were filtered out using EndNote X8 software.

### 2.2. Study Selection

Only RCTs comparing IN ketamine to IV analgesics (morphine, fentanyl, and ketamine) or IN placebo in acute pain management were included. The SRMA’s inclusion criteria were as follows: (1) RCT; (2) population: adults (age ≥ 15 years old) who presented to ED with acute pain with numeric rating scale (NRS) or visual analogue scale (VAS) ≥ 5; (3) intervention: IN ketamine only; (4) comparison: IV morphine, IV fentanyl, IV ketamine or IN placebo; (5) outcomes: pain score reduction, the requirement of rescue analgesia and adverse events. We restricted the publications to the English language only. Ketamine’s analgesic effects on perioperative pain (i.e., subacute pain) following anesthesia, cancer-related pain, and other non-acute pain were eliminated from the study. Studies performed in non-ED settings, such as postoperative or prehospital, were also excluded. We excluded studies where IN ketamine was given as a combination therapy with other analgesics such as inhaled nitrous oxide or IV morphine. Studies that used higher dosage for procedural sedation or IV, IM, oral, or topical routes as ketamine intervention were not considered. Our SRMA eliminated studies that compared IN ketamine to analgesics administered orally, topically, IM, or IN. The titles and abstracts were screened, and eligible RCTs were selected by two authors independently (Y.S.S. and M.A.I.). We gathered full-text copies of the relevant articles to determine if they met the inclusion criteria. Discrepancies between the authors were resolved through discussions.

### 2.3. Outcome Measures

Our primary outcome was to evaluate the analgesic efficacy of IN ketamine in pain score reduction using validated pain scales (NRS or VAS) from baseline to post-intervention and the requirement for rescue analgesics. The following time points were included: 5, 10, 15, 20, 25, 30, and 60 min. We analysed the prevalence of the adverse events, such as dizziness, nausea, vomiting, agitation, difficulty in concentration, confusion, emergence phenomenon, dry mouth, fatigue, disorientation, drowsiness, euphoria, headache, and hypotension as the secondary outcomes for evaluating the safety of IN ketamine.

### 2.4. Data Extraction and Quality Assessment

The data were extracted independently by two authors (Y.S.S. and M.A.I.) from the full-text reports and supplemental materials. We extracted the following information into a data extraction form: study design, study size, study population, causes of pain, intervention details (doses and timing), outcomes of interest, including pain scores pre- and post-intervention, the need for rescue analgesics, and the prevalence of adverse events from each of the eligible study. We attempted contacting the corresponding authors of included trials that had missing or incomplete data. Disagreements were resolved through the discussion among the authors. Two authors (Y.S.S. and M.A.I.) independently assessed the quality of included RCTs using the Joanna Briggs Institute (JBI) critical appraisal tool [24]. The studies were classified as poor quality (high risk of bias), moderate quality (moderate risk of bias), or high quality (low risk of bias) if the total score was ≤49%, 50–69%, or ≥70% [25,26].

### 2.5. Data Synthesis and Analysis

We calculated the mean differences (MDs) with associated 95% confidence intervals (CIs) for the continuous data of pain score reduction at 5, 10, 15, 20, 25, 30, and 60 min of post-intervention for IN ketamine versus IV analgesics (morphine, fentanyl, or ketamine) or placebo. Subgroup analyses were carried out based on the comparative drugs. We computed the odds ratios (ORs) with associated 95% CIs for rescue analgesia and adverse events for the dichotomous data. We also calculated the prevalence of adverse events with associated 95% CIs. We attempted to contact the authors for the unreported outcomes of interest, such as MD or standard deviations. We used the Review Manager (RevMan) calculator to impute missing data when we could not contact the authors as recommended by the Cochrane handbook [27]. We also extracted data from figures or graphs using WebPlotDigitizer Software 4.4 version. RevMan version 5.4 and metaprop codes in the meta (version 4.15-1) and metafor (version 2.4-0) packages of R (version 3.6.3) in RStudio (version 1.3.1093) were used to create all analyses and plots [28].

The random-effects model was used in all meta-analyses, and the *I*^2^ statistic was used to assess trial heterogeneity (*I*^2^ > 75% indicating significant heterogeneity). The Cochran’s Q test was employed to assess the significance of the heterogeneity test; a *p*-value of 0.05 indicated significant heterogeneity [29,30]. We performed sensitivity analysis by excluding the low or moderate quality studies and using a fixed-effects model. We planned to perform publication bias analysis if there were 10 or more studies.

## 3. Results

### 3.1. Study Selection

**Figure 1:**
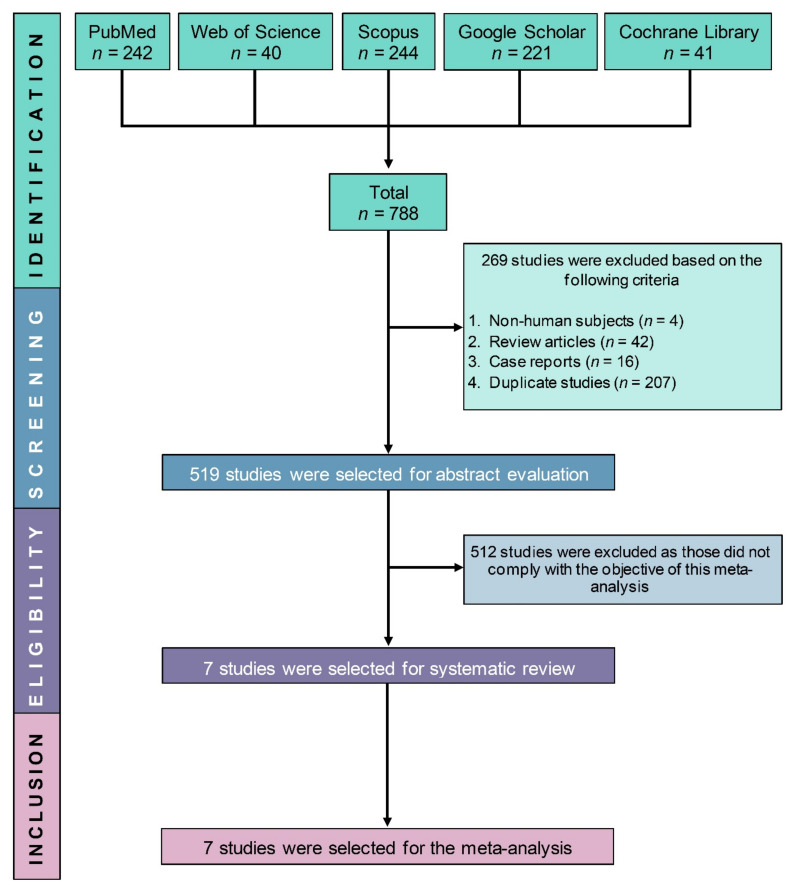
PRISMA flow diagram of study selection.

Our search via the electronic databases identified 788 potentially relevant studies. After removing 269 studies (non-human studies (*n* = 4), review articles (*n* = 42), case reports (*n* = 16), duplicate studies (*n* = 207)), another 512 studies were excluded based on their abstracts as they did not comply with the objective and inclusion criteria of this study. Finally, seven RCTs [20,22,31,32,33,34,35] with a total of 1760 participants were included in this SRMA (Figure 1).

### 3.2. Study Characteristics

The seven included trials involving 1760 participants; 883 participants being randomised to receive IN ketamine, whereas 877 participants being randomised to receive IV analgesics (i.e., morphine, fentanyl, or ketamine) or placebo. All trials were double-blinded RCTs with mean ages ranging from 30.4 to 40.3 years. Five trials were conducted in Iran [20,31,32,33,34], and another two trials were conducted in Tunisia [22] and Israel [35]. Causes of acute pain included acute traumatic pain [20,22,34,35] and renal colic [31,32,33]. In six trials [20,22,32,33,34,35], researchers used the VAS as a pain scale, whereas only a single trial used the NRS [31]. All the major characteristics of the included studies are presented in Table 1.

### 3.3. Study Quality

The detailed quality assessment of the included studies is shown in Table S2. Briefly, 71.4% of the included studies [20,22,31,32,33] were of high-quality (low-risk of bias), and the remaining two studies [34,35] were of moderate-quality (28.6%). None of the studies was low-quality studies (high-risk of bias).

### 3.4. Primary Outcomes (Efficacy of IN Ketamine)

#### 3.4.1. Pain Reduction from Baseline

**Figure 2:**
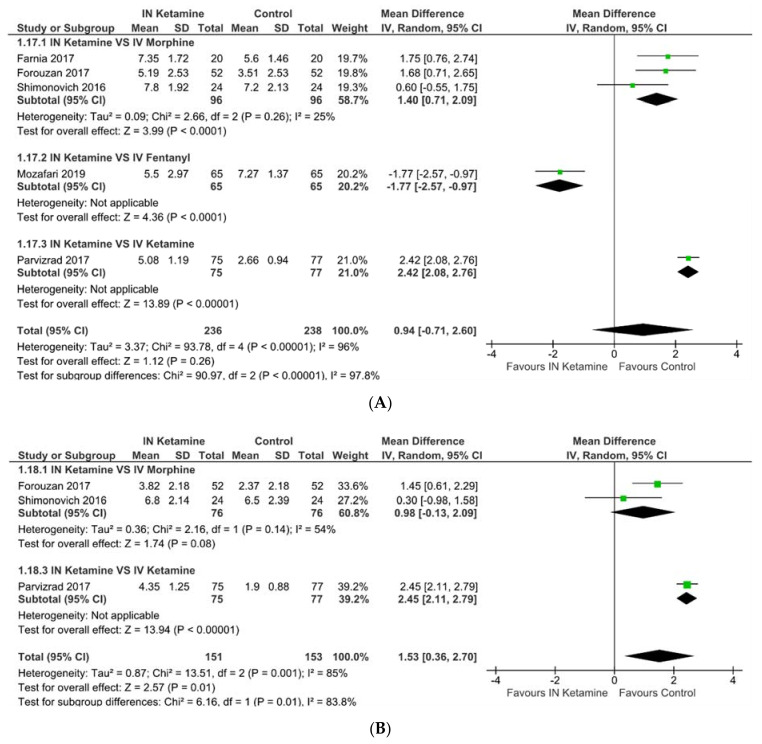

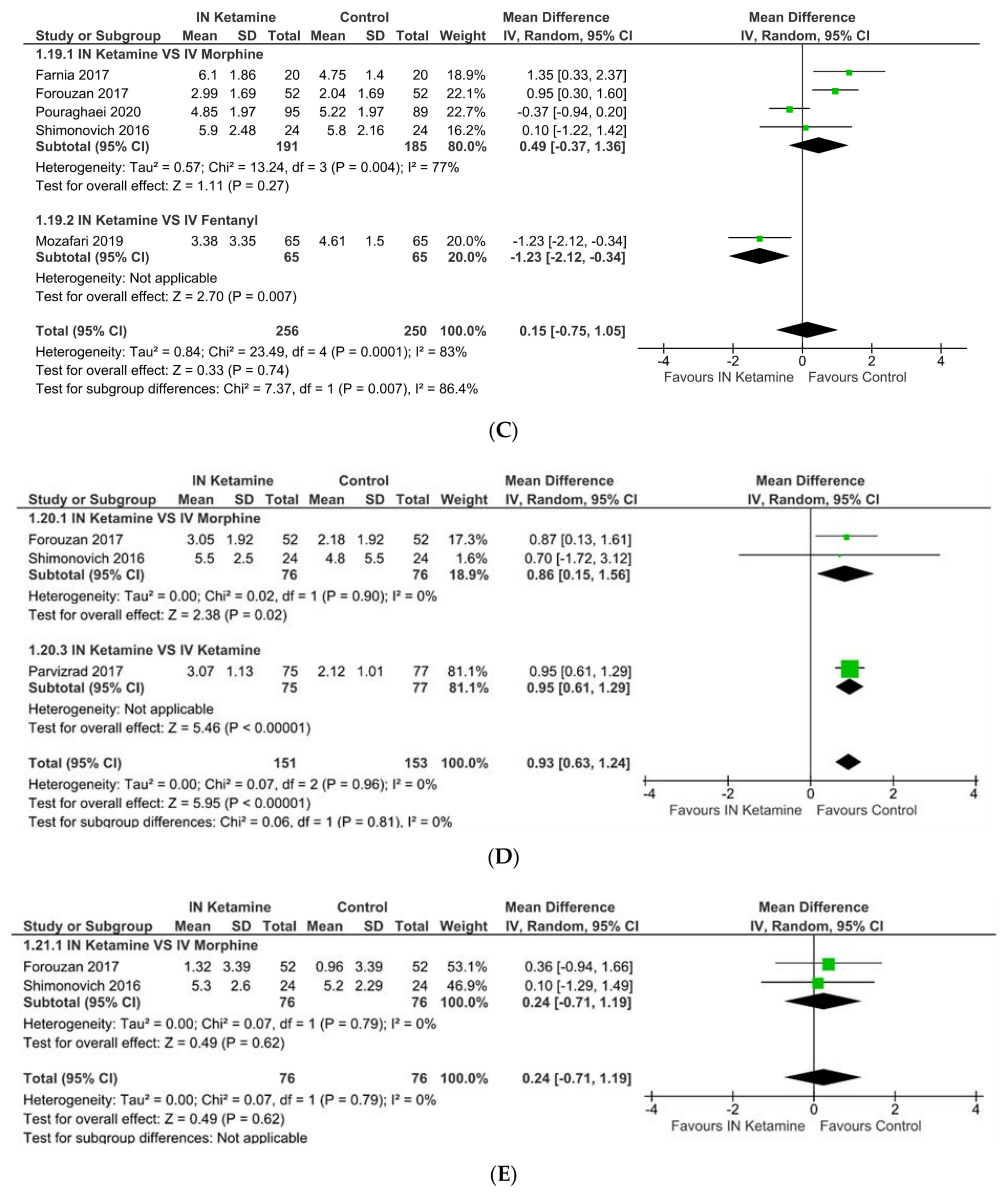

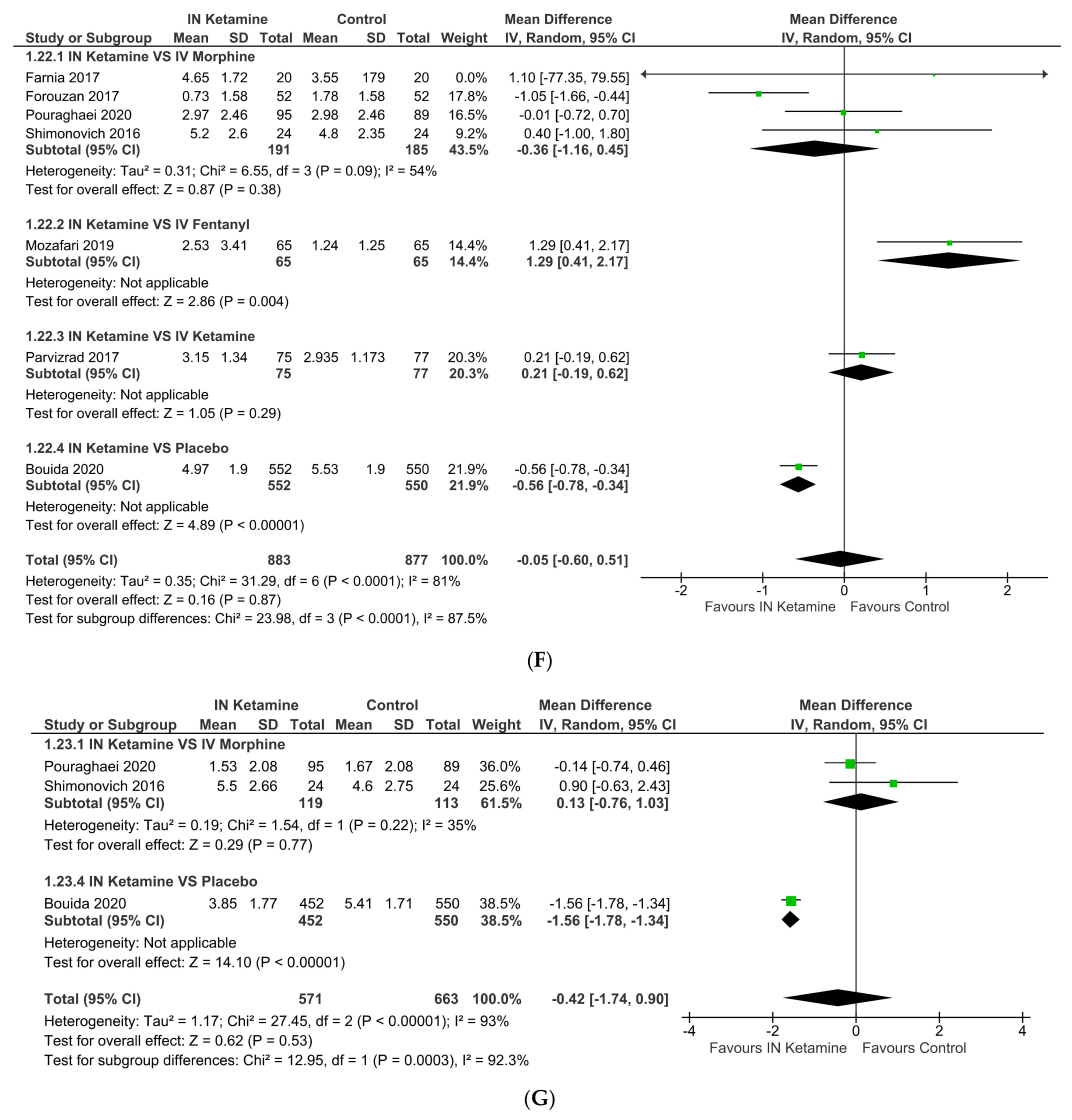
Change in pain score at (**A**) 5 min, (**B**) 10 min, (**C**) 15 min, (**D**) 20 min, (**E**) 25 min, (**F**) 30 min, and (**G**) 60 min.

Comparing IN ketamine with control group (IV analgesics or placebo), we found no statistically significant differences in pain scores at 5 (MD 0.94, *p* = 0.26), 15 (MD 0.15, *p* = 0.74), 25 (MD 0.24, *p* = 0.62), 30 (MD −0.05, *p* = 0.87), and 60 min (MD −0.42, *p* = 0.53) (Figure 2). A pooled analysis of four trials comparing IN ketamine with IV morphine showed no statistically significant difference in pain score at 15 min (MD 0.49, *p* = 0.27) and 30 min (MD −0.36, *p* = 0.38).

#### 3.4.2. Requirements of Rescue Analgesics

**Figure 3:**
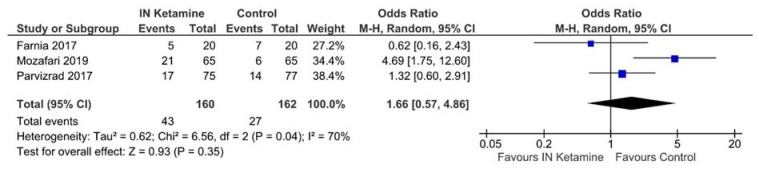
The need for rescue analgesics.

Three trials investigated the need for rescue analgesics [20,32,33]. When comparing IN ketamine to IV analgesics, there was no significant difference in the need for rescue analgesics (OR 1.66, 95% CI: 0.57−4.86, *p* = 0.35, *I*^2^ = 70%; Figure 3).

### 3.5. Secondary Outcomes (Safety of IN Ketamine)

#### Adverse Events

**Figure 4:**
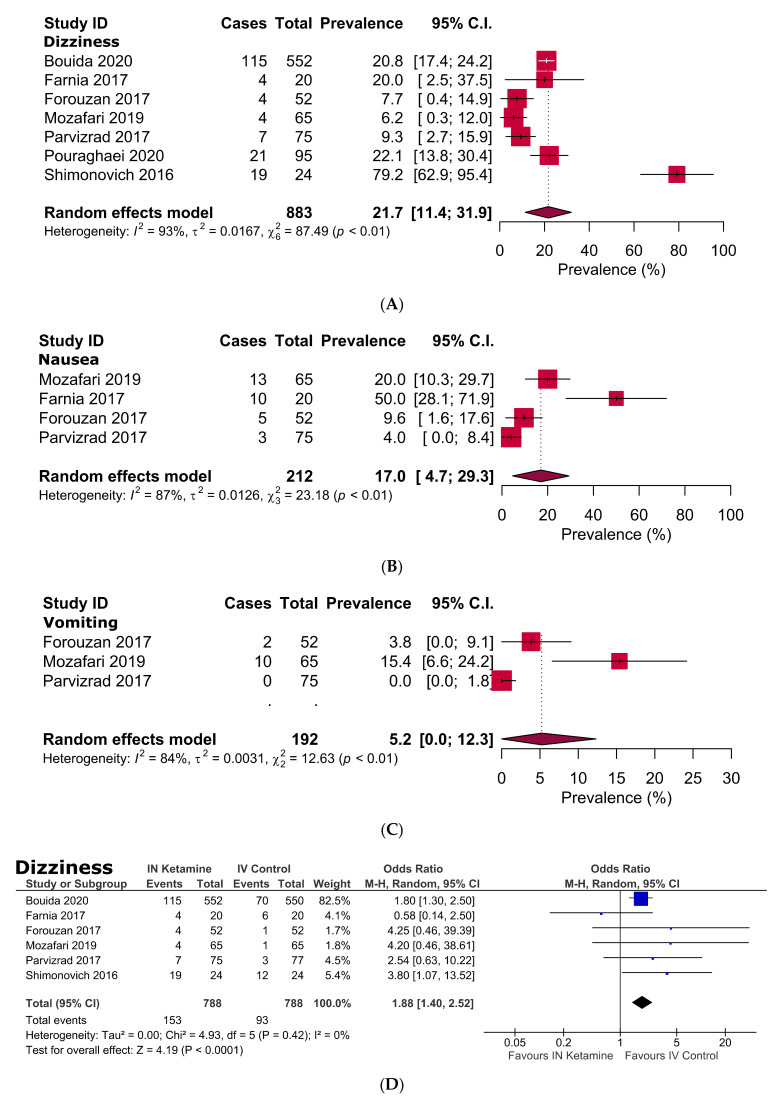

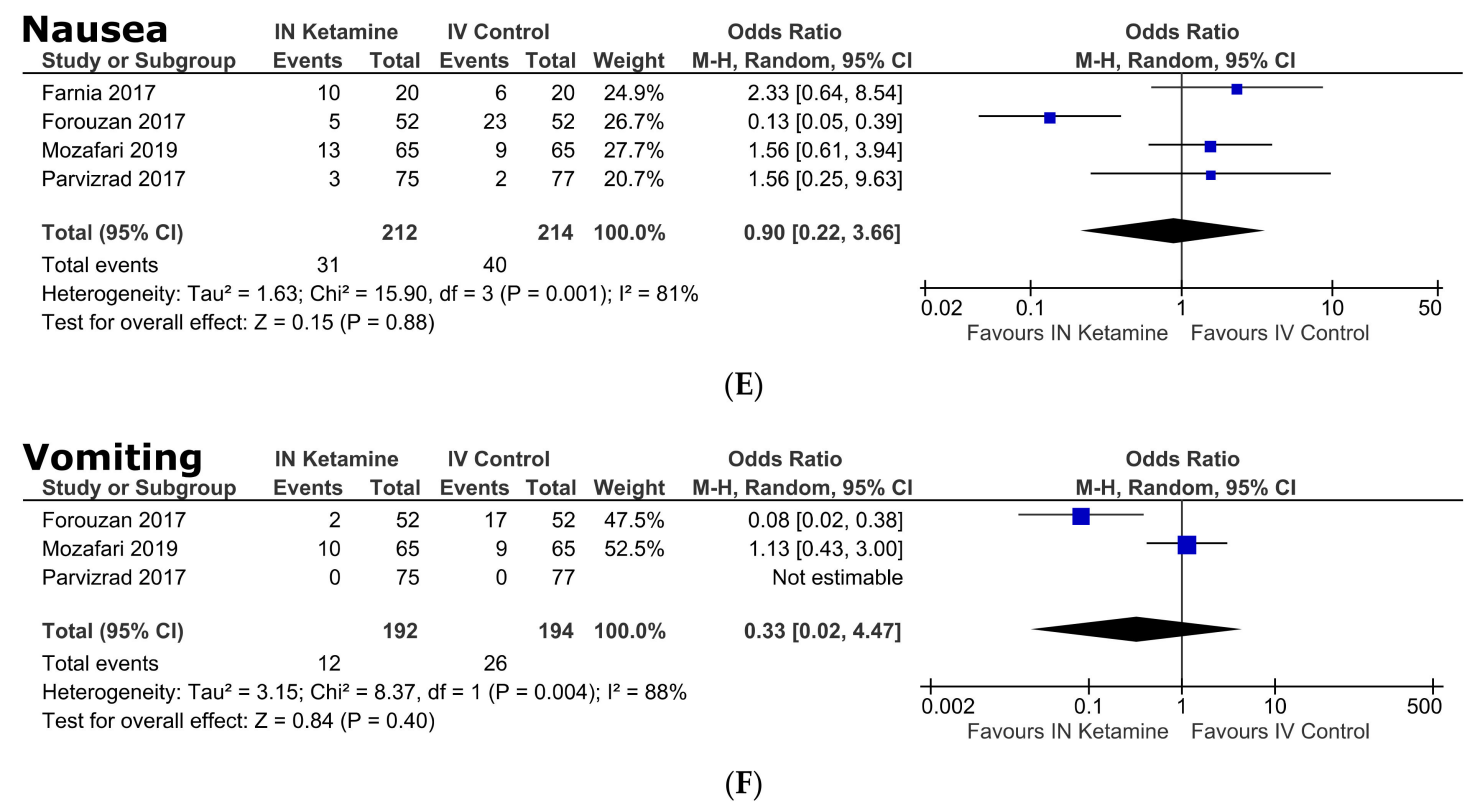
Prevalence (**A**–**C**) and odds ratio (**D**–**F**) of dizziness, nausea and vomiting in acute pain patients following administration of IN ketamine in emergency settings.

Detailed adverse events of the included studies are shown in Table 2 and Figure S1. Some mild adverse effects were observed in patients who received IN ketamine. For instance, in IN ketamine group, the odds of dizziness (OR 1.9, 95% CI: 1.4–2.5, *p* < 0.0001), difficulty concentrating (OR 5.3, 95% CI: 1.5–19.0, *p* = 0.01), confusion (OR 7.0, 95% CI: 1.6–29.9, *p* = 0.009), and disorientation (OR 9.2, 95% CI: 3.6–23.4, *p* < 0.00001) were significantly higher than that of the control group. For hypotension, the odds were significantly lower in the IN ketamine group than the control group (OR 0.04, 95% CI: 0.0–0.68, *p* = 0.03); besides, for other adverse events, there was no significant difference between IN ketamine and the control group (Table 2 and Figure S1). The pooled prevalence of the adverse events in adults receiving IN ketamine demonstrated that dizziness, nausea, difficulty concentrating, confusion, emergence phenomenon, and dry mouth were experienced by 21.7%, 17.0%, 58.3%, 50.0%, 30.0%, and 25.0%, respectively (Table 2, Figure 4 and Figure S1).

### 3.6. Sensitivity Analyses

In the sensitivity analyses excluding low- or medium-quality studies and using a fixed-effects model, only minor differences (ranging from 0.45 lower to 0.92 higher) were observed estimating pain score and rescue analgesic requirement compared to the main findings (Table 3).

## 4. Discussion

Ketamine has been used globally as an anesthetic agent for more than 50 years and is widely used in ED for procedural sedation and as a RSI induction agent [36,37]. Multiple studies have been focused on ketamine utilisation and suggested that low-dose-ketamine (LDK) can be used alone or as an adjuvant analgesic for effective and safe acute pain relief [38,39,40,41]. Opioids are the mainstay of analgesic therapy for moderate to severe pain in ED settings. However, the available data showed a significant correlation between the frequency of opioids prescriptions and the death secondary to opioids use or abuse. This is mainly due to the effects of opioids such as dependence, tolerance, suppression of respiratory center and hypotension, especially in opioid overdose [10,11]. Hence, according to the 2017 policy statement ACEP, acute pain management should begin with a non-opioid drug [12].

One of the common reasons for delays in pain management at EDs is due to difficult IV access. A prospective cohort study by Witting found that 39% of adult patients in ED had a failed first attempt of IV access, and 22% of them also had a failed second attempt [42]. Although available data regarding the usage of IN ketamine are limited, the IN administration of ketamine could be appealing for pain control, especially in overcrowded Eds, because it can be delivered easier, faster and efficient with fewer adverse effects. IN ketamine has been shown in a few trials to be effective and safe as an analgesic agent in burn dressing [43], in post-surgical pain [44], and pediatric laceration repair [45]. An observational study by Yeaman et al. [46] discovered that 1 mg/kg of IN ketamine was moderately effective as a single agent in relieving severe pain among adult patients in an ED setting. Another observational study by Andolfatto et al. [47] reported that IN ketamine significantly reduced the VAS pain scores in 88% of ED patients. More recent research by the same research group in a prehospital setting added that inhaled nitrous oxide could exert significant pain reduction at 30 min in patients receiving IN ketamine patients (76%) compared to patients receiving placebo (41%) [48].

In our SRMA, IN ketamine was found to be non-inferior compared to IV analgesics as the analyses showed no significant differences in pain scores at 5, 15, 25, 30, and 60 min post-intervention among adults in the ED. Interestingly, we found better pain reduction with IV analgesics at 10 and 20 min post-intervention. Such inconsistent results in our analysis were primarily influenced by the trial of Parvizrad et al., which reported IN ketamine only achieved equal analgesic effect as IV ketamine at 30 min post-intervention in a population of acute traumatic pain [20]. This is possibly due to the delayed onset of action of IN ketamine (10 min) as compared with IV ketamine (10 s) [18]. Comparing our analysis with the pediatric population, the SRMA by Oliveira et al. [49] observed no significant difference in pain scores between IN ketamine and IN fentanyl at all different time points post-intervention. The consistent results in their SRMA can be attributed to uniform reporting of data and similar comparator groups in all trials.

On pooled analysis comparing IN ketamine with IV morphine, we found no statistically significant difference in pain reduction at 10, 15, 25, 30, and 60 min. However, we noticed IV morphine is more efficient than IN ketamine in pain reduction at 5 min. This is possibly due to a delayed onset of IN ketamine compared with IV morphine in pain reduction. It is consistent with a systematic review by Poonai et al. [50], which reported the means onset of IN ketamine for sedation ranging from 3.6 to 11.6 min.

Reduced use of opioids as rescue analgesia is desirable due to the current opioid crisis in the United States [51]. In our meta-analysis, the rescue analgesics requirement for adults who received IN ketamine showed no significant increment compared with IV analgesics. It yielded a wide CI, which implies that larger trials are required to evaluate the impact of IN ketamine on rescue analgesics. Bouida et al. [22] demonstrated a significantly lower requirement of opioids in IN ketamine group compared to the placebo group. However, for our rescue analgesic requirement analysis, this RCT was excluded to minimise the heterogeneity due to the placebo usage compared to IN Ketamine.

The safety of analgesics in EDs is as crucial as their efficacy. Therefore, analgesics with less adverse effects for pain control are desired, especially in EDs due to staff shortage, limited beds and suboptimal patient monitoring in overcrowded EDs [5]. No life-threatening adverse events associated with LDK were reported in a few SRMAs [39,40,41]. An SRMA by Oliveira et al. also revealed no serious adverse event associated with IN ketamine compared to IN fentanyl [49]. This is consistent with our study, in which none of the included trials reported any severe adverse events with IN ketamine. However, a single trial reported higher hypotension incidents with IV morphine than IN ketamine [33].

In our current study, the common non-severe and transient adverse events associated with IN ketamine were dizziness, nausea, difficulty concentrating, confusion, emergence phenomenon, and dry mouth. This is in line with a systematic review of side effects associated with ketamine use in depression, where psychotomimetic side effects, neurological or cognitive effects, and other side effects related to the gastrointestinal, ocular, respiratory, and urological systems were found in 42%, 23%, and 23% of the studies, respectively [52]. Dizziness and nausea caused by IN ketamine are most likely due to NMDA receptor blockade in the vestibular system [53,54]. The cognitive impairment such as difficulty concentrating or confusion and the psychotomimetic effect such as emergence phenomenon associated with ketamine are due to the NMDA receptor blockade effect and reduction of presynaptic release of glutamate in CNS [14,55]. Additionally, the antimuscarinic effect of ketamine is the possible cause of dry mouth [14,56]. Studies by Murrough et al. reported LDK use in patients with treatment-resistant depression were associated with transient mild acute neurocognitive effects. They also found no persistent neurocognitive effects beyond seven days after treating LDK [57,58].

An SRMA by Balzer et al. compared IV LDK and IV morphine for acute pain control in the ED showed equivalent analgesic efficacy within 60 min of administration with similar safety profiles [59]. This is consistent with our analysis suggesting that IN ketamine can be considered a single alternative agent for acute pain management in ED.

There are several strengths noted in our study. Firstly, this SRMA only included RCTs representing a high level of evidence. An exhaustive search of the literature was performed via multiple databases to ensure the thorough identification of relevant studies. The stringent exclusion criteria of our study avoided confounding factors such as population with altered pain physiology and altered drugs metabolisms (i.e., chronic cancer pain, post-surgical pain, or pediatric population) which would affect the accuracy of our results. Based on the quality assessment, 71.4% of the included studies were high-quality (low-risk of bias), ensuring reliable results. Recently, Li et al. [60] published an SRMA on the efficacy of IN ketamine for acute pain. Nevertheless, our PROSPERO-registered SRMA is superior since it focused exclusively on the analgesic efficacy of IN ketamine by excluding the RCTs in which IN ketamine was used in combination with other analgesics such as inhaled nitrous oxide [48] or IV morphine [61]. Additionally, we conducted sensitivity analyses by excluding the low or moderate quality studies and using a fixed-effects model as this is an important strategy to ensure the robustness and reliability of the generated results. Furthermore, due to our exhaustive search of the literature, we included two additional RCTs [20,34] and this allowed us to analyze the need for rescue analgesics between IN ketamine and IV analgesics. We also conducted four subgroup analyses. Whereas subgroup analysis and comparison of the requirement of rescue analgesics between IN ketamine and IV analgesics were absent in the SRMA conducted by Li et al. Overall, our study produced intriguing results for the ED clinicians.

We were aware of few limitations in our SRMA. Firstly, only seven trials fulfilled our inclusion criteria. As a result, more clinical trials are needed to assess the efficacy and safety of IN ketamine for acute pain management among adults in ED. Secondly, some of the data might be less precise as we used WebPlotDigitizer Software to extract data from figures and RevMan calculator to impute the missing data as recommended by the Cochrane handbook as we failed to contact the authors. Thirdly, based on the JBI critical appraisal tool, we had 71.4% high-quality (low-risk of bias), 28.6% moderate-quality (28.6%), and none of the studies was low-quality. However, this tool does not thoroughly assess the studies quality (i.e., study design—superiority, non-inferiority, or equivalence and methods of sample size calculation). Finally, heterogeneity among studies was significant, probably due to limited trials with variations in populations and different control groups.

## 5. Conclusions

In conclusion, this SRMA revealed that IN ketamine is non-inferior compared to IV analgesics for acute pain management among adults at 5, 15, 25, 30, and 60 min post-intervention in the ED setting and do not require higher rescue analgesics. The favorable safety profile of IN ketamine as no reported life-threatening adverse event also encourages IN ketamine as a pain reliever in the EDs. However, insufficient evidence proves that IN ketamine is superior to IV analgesics in acute pain management. Therefore, more RCTs are required to evaluate further the efficacy and safety of IN ketamine in managing acute pain in the EDs.

## Figures and Tables

**Table 1:** Major characteristics of the included studies.

Study ID	Country	Sample Size [Ketamine (% Female)/ Control (% Female)]	Age in Years (Mean ± SD)	Causes of Acute Pain	Pain Scale Used	Intervention	Comparator	Outcomes
						Route of Administration, Dose		
Bouida 2020	Tunisia	552 (59.6%)/ 550 (58.5%)	37.2 ± 12.8	Acute traumatic pain	VAS	IN Ketamine, 50 mg	IN Placebo	VAS at 15, 30, 60, and 120 min Requirement for rescue analgesia (opioid: IV Morphine 0.1mg/kg/SC Tramadol 100mg or non-opioid: IV Paracetamol 1 g/IV Ketoprofen 100 mg) Adverse events % of patients discharged from ED with VAS < 30
Pouraghaei 2020	Iran	95 (NR)/ 89 (NR)	40.3 ± 4.6	Renal colic	NRS	IN Ketamine, 1 mg/kg and IV Placebo	IV Morphine, 0.1 mg/kg and IN Placebo	NRS at 15, 30, and 60 min Adverse events
Mozafari 2019	Iran	65 (NR)/ 65 (NR)	36.9 ± 10.6	Renal colic	VAS	IN Ketamine, 1 mg/kg and IV Placebo	IV Fentanyl, 1 μg/kg and IN Placebo	VAS at 5, 15, and 30 min Requirement for rescue analgesia (IV Morphine 0.1 mg/kg) Adverse events
Farnia 2017	Iran	20 (40%)/ 20 (15%)	37.0 ± 11.3	Renal colic	VAS	IN Ketamine, 1 mg/kg and IV Placebo	IV Morphine, 0.1 mg/kg and IN Placebo	VAS at 5, 15, and 30 min Requirement for rescue analgesia (IV Fentanyl infusion 1–2 μg/kg, every 5 min and titrated) Adverse events
Forouzan 2017	Iran	52 (28.8%)/ 52 (17.3%)	30.4 ± 11.5	Acute traumatic pain	VAS	IN Ketamine, 1 mg/kg	IV Morphine, 0.1 mg/kg	VAS at 5, 10, 15, 20, 25, and 30 min Time of onset of VAS reduction Adverse events Change of hemodynamic parameters
Parvizrad 2017	Iran	75 (41.6%)/ 77 (59.8%)	34.6 ± 12.2	Acute traumatic pain	VAS	IN Ketamine, 0.4 mg/kg and IV Placebo	IV Ketamine, 0.2 mg/kg and IN Placebo	VAS at 5, 10, 20, and 30 min Requirement for rescue analgesia (low dose IV Morphine) Adverse events Patient’s satisfaction
Shimonovich 2016	Israel	24 (29.2%)/ 24 (25%)	39.4 ± NR	Acute traumatic pain	VAS	IN Ketamine, 1 mg/kg	IV Morphine, 0.1 mg/kg	Time to achievement of VAS reduction ≥ 15 mm Maximum pain reduction Time to maximum pain reduction VAS at 5, 10, 15, 20, 25, 30, 35, 40, 45, 50, 55, and 60 min Patient’s satisfaction Adverse events Change of hemodynamic parameters

VAS: Visual analog scale, NRS: Numerical rating scale, IN: Intranasal, IV: Intravenous, SC: Subcutaneous.

**Table 2:** Adverse events.

Adverse Effects	Prevalence of Adverse Events [95% CIs] (%)	Number of Studies Analysed	Total Number of Patients	Heterogeneity		OR [95% CIs]	*p*-Value	Heterogeneity	
				*I* ^2^	*p*-Value			*I* ^2^	*p*-Value
Dizziness	21.7 [11.4–31.9]	7	883	93%	<0.0001	1.9 [1.4–2.5] *	<0.0001	0%	0.42
Nausea	17.0 [4.7–29.3]	4	212	87%	<0.0001	1.0 [0.2–3.7]	0.88	81%	0.001
Vomiting	5.2 [0.0–12.3]	3	192	84%	0.003	0.3 [0.0–4.5]	0.40	88%	0.004
Agitation	2.0 [0.0–4.3]	2	140	0%	0.63	2.1 [0.3–16.8]	0.47	0%	0.43
Difficulty concentrating	58.3 [38.6–78.1]	1	24	NA	NA	5.3 [1.5–19.0]	0.01	NA	NA
Confusion	50 [30.0–70.0]	1	24	NA	NA	7.0 [1.6–29.9]	0.009	NA	NA
Emergence phenomenon	30.0 [9.9–50.1]	1	20	NA	NA	18.4 [1.0–352.6]	0.05	NA	NA
Dry mouth	25.0 [7.7–42.3]	1	24	NA	NA	0.09 [0.02–0.3]	0.0004	NA	NA
Fatigue	14.7 [6.7–22.7]	1	75	NA	NA	0.9 [0.4–2.0]	0.71	NA	NA
Disorientation	7.8 [5.6–10.0]	1	552	NA	NA	9.2 [3.6–23.4]	<0.00001	NA	NA
Drowsiness	10.7 [3.7–17.7]	1	75	NA	NA	3.0 [0.8–11.6]	0.12	NA	NA
Euphoria	10.7 [3.7–17.7]	1	75	NA	NA	0.5 [0.2–1.4]	0.19	NA	NA
Headache	4.6 [0.0–9.7]	1	65	NA	NA	3.1 [0.3–30.6]	0.33	NA	NA
Hypotension	0.0 [0.0–6.5]	1	20	NA	NA	0.04 [ 0.0–0.7]	0.03	NA	NA

CIs: Confidence intervals; OR: Odds ratio, NA: Not applicable. * Six studies were considered in OR analysis.

**Table 3:** Sensitivity analyses.

Strategies of Sensitivity Analyses	Risk Ratio or Mean Difference [95% CIs] (%)	Number of Studies Analysed	Difference of Results	Total Number of Subjects	Heterogeneity	
					*I* ^2^	*p*-Value
**Pain score (5 min)**						
Excluding low- or medium-quality studies	0.81 [−1.84, 3.46]	3	0.13 lower	322	98%	<0.00001
Using a fixed-effects model	1.69 [1.41–1.97]	5	0.75 higher	474	96%	<0.00001
**Pain score (10 min)**						
Excluding low- or medium-quality studies	2.45 [2.11–2.79]	1	0.92 higher	152	NA	NA
Using a fixed-effects model	2.19 [1.88–2.50]	3	0.66 higher	304	85%	0.0001
**Pain score (15 min)**						
Excluding low- or medium-quality studies	−0.12 [−1.37–1.14]	3	0.27 lower	354	86%	0.0008
Using a fixed-effects model	0.08 [−0.27–0.44]	5	0.07 lower	506	82%	0.0001
**Pain score (20 min)**						
Excluding low- or medium-quality studies	0.95 [0.61, 1.29]	1	0.02 higher	152	NA	NA
Using a fixed-effects model	0.93 [0.63–1.24]	3	No change	304	0%	0.96
**Pain score (25 min)**						
Using a fixed-effects model	0.24 [−0.71–1.19]	2	No change	152	0%	0.79
**Pain score (30 min)**						
Excluding low- or medium-quality studies	0.16 [−0.46-0.78]	5	0.19 higher	1608	73%	0.005
Using a fixed-effects model	−0.13 [−0.37–0.11]	7	0.10 lower	1760	76%	0.0003
**Pain score (60 min)**						
Excluding low- or medium-quality studies	−0.85 [−2.24–0.54]	2	0.45 lower	1186	91%	0.001
Using a fixed-effects model	−0.73 [−1.14, −0.32]	3	0.33 lower	1234	87%	0.0005
**Rescue analgesic requirement**						
Excluding low- or medium-quality studies	1.66 [0.57–4.86]	3	No change	322	70%	0.04
Using a fixed-effects model	1.82 [1.06–3.12]	3	0.16 higher	322	70%	0.04

CIs: confidence intervals.

## Data Availability

The data presented in this study are available in the main text and Supplementary Materials.

## Supplementary Materials

The following are available online at https://www.mdpi.com/article/10.3390/jcm10173978/s1, Figure S1: Prevalence (A–K) and odds ratio (L–V) of adverse events; Table S1: Search strategies; Table S2: Quality assessment of the included randomised controlled trials.

## References

[1] Chang H.Y., Daubresse M., Kruszewski S.P., Alexander G.C. (2014). Prevalence and treatment of pain in EDs in the United States, 2000 to 2010. Am. J. Emerg. Med..

[2] Samcam I., Papa L. (2016). Acute Pain Management in the Emergency Department. Pain Management.

[3] Rupp T., Delaney K.A. (2004). Inadequate analgesia in emergency medicine. Ann. Emerg. Med..

[4] Sinatra R. (2010). Causes and Consequences of Inadequate Management of Acute Pain. Pain Med..

[5] Kahsay D.T., Pitkäjärvi M. (2019). Emergency nurses´ knowledge, attitude and perceived barriers regarding pain Management in Resource-Limited Settings: Cross-sectional study. BMC Nurs..

[6] Todd K.H., Ducharme J., Choiniere M., Crandall C.S., Fosnocht D.E., Homel P., Tanabe P. (2007). Pain in the emergency department: Results of the pain and emergency medicine initiative (PEMI) multicenter study. J. Pain.

[7] Vazirani J., Knott J.C. (2012). Mandatory pain scoring at triage reduces time to analgesia. Ann. Emerg. Med..

[8] Schwartz R.B., Charity B.M. (2001). Use of night vision goggles and low-level light source in obtaining intravenous access in tactical conditions of darkness. Mil. Med..

[9] Hedegaard H., Miniño A.M., Warner M. Drug Overdose Deaths in the United States, 1999–2017. 2018. NCHS Data Brief (Accession Number: CDC:84647). https://stacks.cdc.gov/view/cdc/84647.

[10] Alexander G.C., Kruszewski S.P., Webster D.W. (2012). Rethinking opioid prescribing to protect patient safety and public health. JAMA.

[11] Campbell W. (2012). Guide to prescribing in today’s management of severe pain. Prescriber.

[12] American College of Emergency Physicians (2017). Optimizing the Treatment of Acute Pain in the Emergency Department. Ann. Emerg. Med..

[13] Li L., Vlisides P.E. (2016). Ketamine: 50 Years of Modulating the Mind. Front. Hum. Neurosci..

[14] Pai A., Heining M. (2007). Ketamine. BJA Educ..

[15] Ahern T.L., Herring A.A., Anderson E.S., Madia V.A., Fahimi J., Frazee B.W. (2015). The first 500: Initial experience with widespread use of low-dose ketamine for acute pain management in the ED. Am. J. Emerg. Med..

[16] Hirlinger W.K., Dick W. (1984). Intramuscular ketamine analgesia in emergency patients. II. Clinical study of traumatized patients. Anaesthesist.

[17] Green S.M., Roback M.G., Kennedy R.M., Krauss B. (2011). Clinical practice guideline for emergency department ketamine dissociative sedation: 2011 update. Ann. Emerg. Med..

[18] Hurth K.P., Jaworski A., Thomas K.B., Kirsch W.B., Rudoni M.A., Wohlfarth K.M. (2020). The Reemergence of Ketamine for Treatment in Critically Ill Adults. Crit. Care Med..

[19] Carr D.B., Goudas L.C., Denman W.T., Brookoff D., Staats P.S., Brennen L., Green G., Albin R., Hamilton D., Rogers M.C. (2004). Safety and efficacy of intranasal ketamine for the treatment of breakthrough pain in patients with chronic pain: A randomized, double-blind, placebo-controlled, crossover study. Pain.

[20] Parvizrad R., Pakniyat A., Malekianzadeh B., Almasi-Hashiani A. (2017). Comparing the analgesic effect of intranasal with intravenous ketamine in isolated orthopedic trauma: A randomized clinical trial. Turk. J. Emerg. Med..

[21] McGuinness S.K., Wasiak J., Cleland H., Symons J., Hogan L., Hucker T., Mahar P.D. (2011). A systematic review of ketamine as an analgesic agent in adult burn injuries. Pain Med..

[22] Bouida W., Bel Haj Ali K., Ben Soltane H., Msolli M.A., Boubaker H., Sekma A., Beltaief K., Grissa M.H., Methamem M., Boukef R. (2020). Effect on Opioids Requirement of Early Administration of Intranasal Ketamine for Acute Traumatic Pain. Clin. J. Pain.

[23] Page M.J., McKenzie J.E., Bossuyt P.M., Boutron I., Hoffmann T.C., Mulrow C.D., Shamseer L., Tetzlaff J.M., Moher D. (2021). Updating guidance for reporting systematic reviews: Development of the PRISMA 2020 statement. J. Clin. Epidemiol..

[24] Moola S., Munn Z., Tufanaru C., Aromataris E., Sears K., Sfetc R., Currie M., Lisy K., Qureshi R., Mattis P. (2020). Chapter 7: Systematic Reviews of Etiology and Risk. Joanna Briggs Institute Reviewer’s Manual.

[25] Islam M.A., Alam S.S., Kundu S., Hossan T., Kamal M.A., Cavestro C. (2020). Prevalence of Headache in Patients with Coronavirus Disease 2019 (COVID-19): A Systematic Review and Meta-Analysis of 14,275 Patients. Front. Neurol..

[26] Chia Y.C., Islam M.A., Hider P., Woon P.Y., Johan M.F., Hassan R., Ramli M. (2021). The Prevalence of *TET2* Gene Mutations in Patients with BCR-ABL-Negative Myeloproliferative Neoplasms (MPN): A Systematic Review and Meta-Analysis. Cancers.

[27] Higgins J.P., Thomas J., Chandler J., Cumpston M., Li T., Page M.J., Welch V.A. (2021). Cochrane Handbook for Systematic Reviews of Interventions Version 6.2 (Updated February 2021).

[28] Viechtbauer W. (2010). Conducting Meta-Analyses in R with the metafor Package. J. Stat. Softw..

[29] Alam F., Islam M.A., Mohamed M., Ahmad I., Kamal M.A., Donnelly R., Idris I., Gan S.H. (2019). Efficacy and Safety of Pioglitazone Monotherapy in Type 2 Diabetes Mellitus: A Systematic Review and Meta-Analysis of Randomised Controlled Trials. Sci. Rep..

[30] Chang C.T., Ang J.Y., Islam M.A., Chan H.K., Cheah W.K., Gan S.H. (2021). Prevalence of Drug-Related Problems and Complementary and Alternative Medicine Use in Malaysia: A Systematic Review and Meta-Analysis of 37,249 Older Adults. Pharmaceuticals.

[31] Pouraghaei M., Moharamzadeh P., Paknezhad S.P., Rajabpour Z.V., Soleimanpour H. (2021). Intranasal ketamine versus intravenous morphine for pain management in patients with renal colic: A double-blind, randomized, controlled trial. World J. Urol..

[32] Mozafari J., Maleki Verki M., Motamed H., Sabouhi A., Tirandaz F. (2020). Comparing intranasal ketamine with intravenous fentanyl in reducing pain in patients with renal colic: A double-blind randomized clinical trial. Am. J. Emerg. Med..

[33] Farnia M.R., Jalali A., Vahidi E., Momeni M., Seyedhosseini J., Saeedi M. (2017). Comparison of intranasal ketamine versus IV morphine in reducing pain in patients with renal colic. Am. J. Emerg. Med..

[34] Forouzan A., Masoumi K., Motamed H., Mozafari J., Gharibi S. (2017). Comparison of intranasal ketamine versus intravenous morphine in pain relief of patient with bone fracture. Int. J. Adv. Biotechnol. Res..

[35] Shimonovich S., Gigi R., Shapira A., Sarig-Meth T., Nadav D., Rozenek M., West D., Halpern P. (2016). Intranasal ketamine for acute traumatic pain in the Emergency Department: A prospective, randomized clinical trial of efficacy and safety. BMC Emerg. Med..

[36] Hopper A.B., Vilke G.M., Castillo E.M., Campillo A., Davie T., Wilson M.P. (2015). Ketamine Use for Acute Agitation in the Emergency Department. J. Emerg. Med..

[37] Pourmand A., Mazer-Amirshahi M., Royall C., Alhawas R., Shesser R. (2017). Low dose ketamine use in the emergency department, a new direction in pain management. Am. J. Emerg. Med..

[38] Lee E.N., Lee J.H. (2016). The Effects of Low-Dose Ketamine on Acute Pain in an Emergency Setting: A Systematic Review and Meta-Analysis. PLoS ONE.

[39] Karlow N., Schlaepfer C.H., Stoll C.R.T., Doering M., Carpenter C.R., Colditz G.A., Motov S., Miller J., Schwarz E.S. (2018). A Systematic Review and Meta-analysis of Ketamine as an Alternative to Opioids for Acute Pain in the Emergency Department. Acad. Emerg. Med..

[40] Yousefifard M., Askarian-Amiri S., Rafiei Alavi S.N., Sadeghi M., Saberian P., Baratloo A., Talebian M.T. (2019). The Efficacy of Ketamine Administration in Prehospital Pain Management of Trauma Patients; a Systematic Review and Meta-Analysis. Arch. Acad. Emerg. Med..

[41] Ghate G., Clark E., Vaillancourt C. (2018). Systematic review of the use of low-dose ketamine for analgesia in the emergency department. CJEM.

[42] Witting M.D. (2012). IV access difficulty: Incidence and delays in an urban emergency department. J. Emerg. Med..

[43] Kulbe J. (1998). The use of ketamine nasal spray for short-term analgesia. Home Healthc. Nurse.

[44] Christensen K., Rogers E., Green G.A., Hamilton D.A., Mermelstein F., Liao E., Wright C., Carr D.B. (2007). Safety and efficacy of intranasal ketamine for acute postoperative pain. Acute Pain.

[45] Tsze D.S., Steele D.W., Machan J.T., Akhlaghi F., Linakis J.G. (2012). Intranasal ketamine for procedural sedation in pediatric laceration repair: A preliminary report. Pediatr. Emerg. Care.

[46] Yeaman F., Meek R., Egerton-Warburton D., Rosengarten P., Graudins A. (2014). Sub-dissociative-dose intranasal ketamine for moderate to severe pain in adult emergency department patients. Emerg. Med. Australas..

[47] Andolfatto G., Willman E., Joo D., Miller P., Wong W.B., Koehn M., Dobson R., Angus E., Moadebi S. (2013). Intranasal ketamine for analgesia in the emergency department: A prospective observational series. Acad. Emerg. Med..

[48] Andolfatto G., Innes K., Dick W., Jenneson S., Willman E., Stenstrom R., Zed P.J., Benoit G. (2019). Prehospital Analgesia with Intranasal Ketamine (PAIN-K): A Randomized Double-Blind Trial in Adults. Ann. Emerg. Med..

[49] Oliveira J.E.S.L., Lee J.Y., Bellolio F., Homme J.L., Anderson J.L. (2020). Intranasal ketamine for acute pain management in children: A systematic review and meta-analysis. Am. J. Emerg. Med..

[50] Poonai N., Canton K., Ali S., Hendrikx S., Shah A., Miller M., Joubert G., Rieder M., Hartling L. (2017). Intranasal ketamine for procedural sedation and analgesia in children: A systematic review. PLoS ONE.

[51] Singh G.K., Kim I.E., Girmay M., Perry C., Daus G.P., Vedamuthu I.P., De Los Reyes A.A., Ramey C.T., Martin E.K., Allender M. (2019). Opioid Epidemic in the United States: Empirical Trends, and A Literature Review of Social Determinants and Epidemiological, Pain Management, and Treatment Patterns. Int. J. Matern. Child Health AIDS.

[52] Short B., Fong J., Galvez V., Shelker W., Loo C.K. (2018). Side-effects associated with ketamine use in depression: A systematic review. Lancet Psychiatry.

[53] Soto E., Flores A., Eróstegui C., Vega R. (1994). Evidence for NMDA receptor in the afferent synaptic transmission of the vestibular system. Brain Res..

[54] Soto E., Vega R. (2010). Neuropharmacology of vestibular system disorders. Curr. Neuropharm..

[55] Newcomer J.W., Farber N.B., Olney J.W. (2000). NMDA receptor function, memory, and brain aging. Dialogues Clin. Neurosci..

[56] Durieux M.E. (1995). Inhibition by ketamine of muscarinic acetylcholine receptor function. Anesth. Analg..

[57] Murrough J.W., Wan L.-B., Iacoviello B., Collins K.A., Solon C., Glicksberg B., Perez A.M., Mathew S.J., Charney D.S., Iosifescu D.V. (2013). Neurocognitive effects of ketamine in treatment-resistant major depression: Association with antidepressant response. Psychopharmacology.

[58] Murrough J.W., Burdick K.E., Levitch C.F., Perez A.M., Brallier J.W., Chang L.C., Foulkes A., Charney D.S., Mathew S.J., Iosifescu D.V. (2015). Neurocognitive effects of ketamine and association with antidepressant response in individuals with treatment-resistant depression: A randomized controlled trial. Neuropsychopharmacology.

[59] Balzer N., McLeod S.L., Walsh C., Grewal K. (2021). Low—Dose ketamine for acute pain control in the emergency department: A systematic review and meta—analysis. Acad. Emerg. Med..

[60] Li X., Hua G., Peng F. (2021). Efficacy of intranasal ketamine for acute pain management in adults: A systematic review and meta-analysis. Eur. Rev. Med. Pharmacol. Sci..

[61] Mohammadshahi A., Abdolrazaghnejad A., Nikzamir H., Safaie A. (2018). Intranasal ketamine administration for narcotic dose decrement in patients suffering from acute limb trauma in emergency department: A double-blind randomized placebo-controlled trial. Adv. J. Emerg. Med..

