# Supplementary material for: Efficacy and Safety of Intranasal Ketamine for Acute Pain Management in the Emergency Setting: A Systematic Review and Meta-Analysis
Source: J Clin Med. 2021 Sep 2;10(17):3978. doi: 10.3390/jcm10173978 (PMC8432265; doi:10.3390/jcm10173978)
Supplement: Supplementary file 1 [file jcm-10-03978-s001.zip › Figure S1_Adverse events.pdf]

A

Study ID	Cases	Total	Prevalence	95% C.I.
**Agitation**				
Mozafari 2019	1	65	1.5	[0; 4.5]
Parvizrad 2017	2	75	2.7	[0; 6.3]
**Random effects model**		**140**	**2.0**	**[0; 4.3]**

Heterogeneity: $I^2 = 0\%$, $\tau^2 = 0$, $\chi^2_1 = 0.22$ ($p = 0.64$)

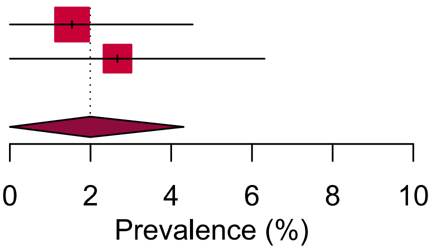

B

Study ID	Cases	Total	Prevalence	95% C.I.
**Difficulty concentrating**				
Shimonovich 2016	14	24	58.3	[38.6; 78.1]

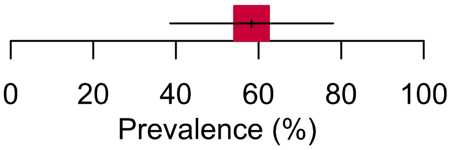

C

Study ID	Cases	Total	Prevalence	95% C.I.
**Confusion**				
Shimonovich 2016	12	24	50.0	[30; 70]

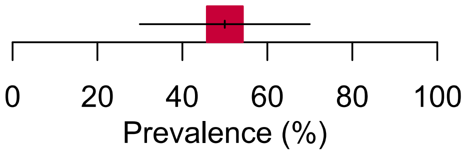

D

Study ID	Cases	Total	Prevalence	95% C.I.
**Emergence phenomenon**				
Farnia 2017	6	20	30.0	[9.9; 50.1]

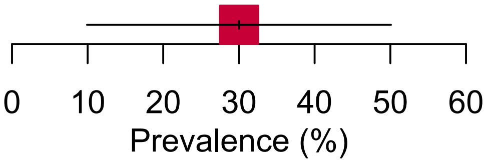

E

Study ID	Cases	Total	Prevalence	95% C.I.
**Dry mouth**				
Shimonovich 2016	6	24	25.0	[7.7; 42.3]

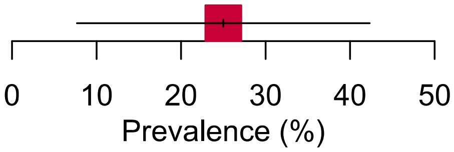

F

Study ID	Cases	Total	Prevalence	95% C.I.
**Fatigue**				
Parvizrad 2017	11	75	14.7	[6.7; 22.7]

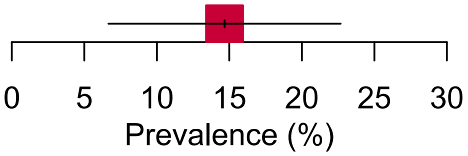

G

Study ID	Cases	Total	Prevalence	95% C.I.
**Disorientation**				
Bouida 2020	43	552	7.8	[5.6; 10]

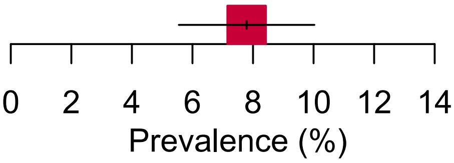

H

Study ID	Cases	Total	Prevalence	95% C.I.
**Drowsiness**				
Parvizrad 2017	8	75	10.7	[3.7; 17.7]

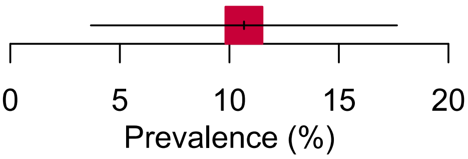

I

Study ID	Cases	Total	Prevalence	95% C.I.
**Euphoria**				
Parvizrad 2017	8	75	10.7	[3.7; 17.7]

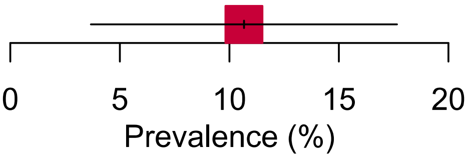

J

Study ID	Cases	Total	Prevalence	95% C.I.
**Headache**				
Mozafari 2019	3	65	4.6	[0; 9.7]

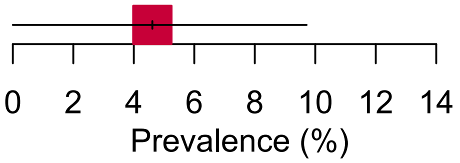

K

Study ID	Cases	Total	Prevalence	95% C.I.
**Hypotension**				
Farnia 2017	0	20	0.0	[0; 6.5]

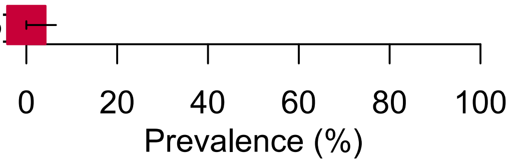

L

Agitation

	IN Ketamine		IV Control			Odds Ratio
Study or Subgroup	Events	Total	Events	Total	Weight	M-H, Random, 95% CI
Mozafari 2019	1	65	1	65	54.4%	1.00 [0.06, 16.34]
Parvizrad 2017	2	75	0	77	45.6%	5.27 [0.25, 111.67]
**Total (95% CI)**		**140**		**142**	**100.0%**	**2.13 [0.27, 16.75]**
Total events	3		1			

Heterogeneity: Tau² = 0.00; Chi² = 0.63, df = 1 (P = 0.43); I² = 0%
Test for overall effect: Z = 0.72 (P = 0.47)

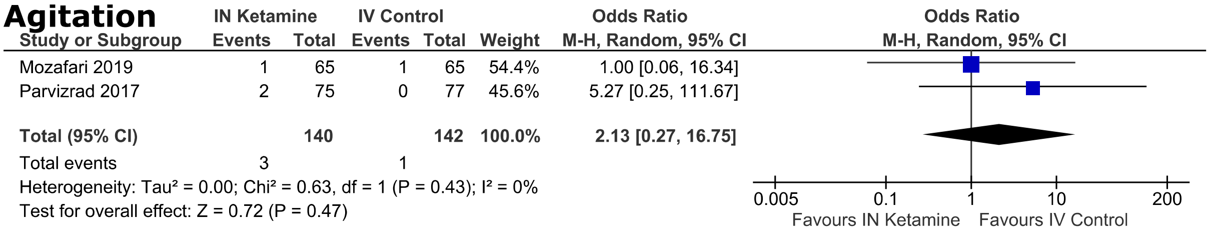

M

Difficulty Concentrating

Study or Subgroup	IN Ketamine Events	IN Ketamine Total	IV Control Events	IV Control Total	Weight	Odds Ratio M-H, Random, 95% CI
Shimonovich 2016	14	24	5	24	100.0%	5.32 [1.48, 19.06]
**Total (95% CI)**		**24**		**24**	**100.0%**	**5.32 [1.48, 19.06]**
Total events	14		5			

Heterogeneity: Not applicable
Test for overall effect: Z = 2.57 (P = 0.01)

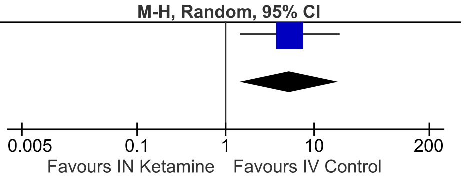

N

Confusion

Study or Subgroup	IN Ketamine Events	IN Ketamine Total	IV Control Events	IV Control Total	Weight	Odds Ratio M-H, Random, 95% CI
Shimonovich 2016	12	24	3	24	100.0%	7.00 [1.64, 29.85]
**Total (95% CI)**		**24**		**24**	**100.0%**	**7.00 [1.64, 29.85]**
Total events	12		3			

Heterogeneity: Not applicable
Test for overall effect: Z = 2.63 (P = 0.009)

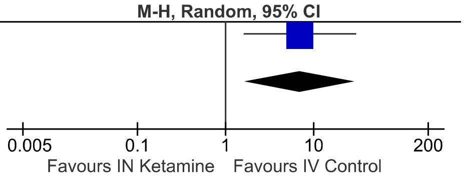

O

Emergence phenomenon

Study or Subgroup	IN Ketamine Events	IN Ketamine Total	IV Control Events	IV Control Total	Weight	Odds Ratio M-H, Random, 95% CI
Farnia 2017	6	20	0	20	100.0%	18.38 [0.96, 352.57]
**Total (95% CI)**		**20**		**20**	**100.0%**	**18.38 [0.96, 352.57]**
Total events	6		0			

Heterogeneity: Not applicable
Test for overall effect: Z = 1.93 (P = 0.05)

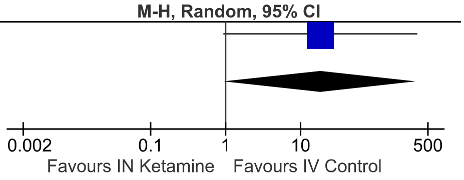

P

Dry Mouth

Study or Subgroup	IN Ketamine Events	IN Ketamine Total	IV Control Events	IV Control Total	Weight	Odds Ratio M-H, Random, 95% CI
Shimonovich 2016	6	24	19	24	100.0%	0.09 [0.02, 0.34]
**Total (95% CI)**		**24**		**24**	**100.0%**	**0.09 [0.02, 0.34]**
Total events	6		19			

Heterogeneity: Not applicable
Test for overall effect: Z = 3.53 (P = 0.0004)

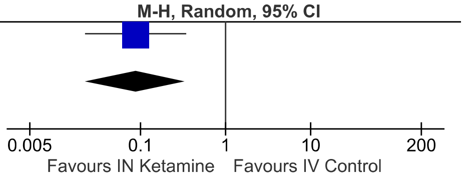

Q

Fatigue

Study or Subgroup	IN Ketamine Events	IN Ketamine Total	IV Control Events	IV Control Total	Weight	Odds Ratio M-H, Random, 95% CI
Parvizrad 2017	11	75	13	77	100.0%	0.85 [0.35, 2.03]
**Total (95% CI)**		**75**		**77**	**100.0%**	**0.85 [0.35, 2.03]**
Total events	11		13			

Heterogeneity: Not applicable
Test for overall effect: Z = 0.37 (P = 0.71)

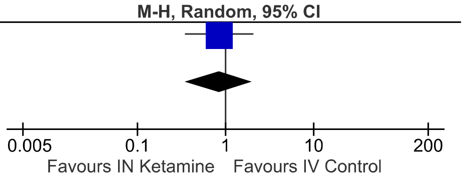

R

Disorientation

Study or Subgroup	IN Ketamine Events	IN Ketamine Total	IV Control Events	IV Control Total	Weight	Odds Ratio M-H, Random, 95% CI
Bouida 2020	43	552	5	550	100.0%	9.21 [3.62, 23.43]
**Total (95% CI)**		**552**		**550**	**100.0%**	**9.21 [3.62, 23.43]**
Total events	43		5			

Heterogeneity: Not applicable
Test for overall effect: Z = 4.66 (P < 0.00001)

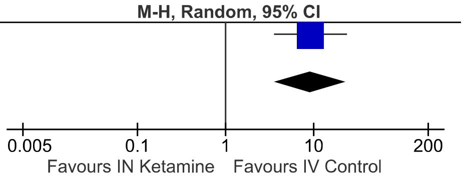

S

Drowsiness

Study or Subgroup	IN Ketamine Events	IN Ketamine Total	IV Control Events	IV Control Total	Weight	Odds Ratio M-H, Random, 95% CI
Parvizrad 2017	8	75	3	77	100.0%	2.95 [0.75, 11.56]
**Total (95% CI)**		**75**		**77**	**100.0%**	**2.95 [0.75, 11.56]**
Total events	8		3			
Heterogeneity: Not applicable						
Test for overall effect: Z = 1.55 (P = 0.12)						

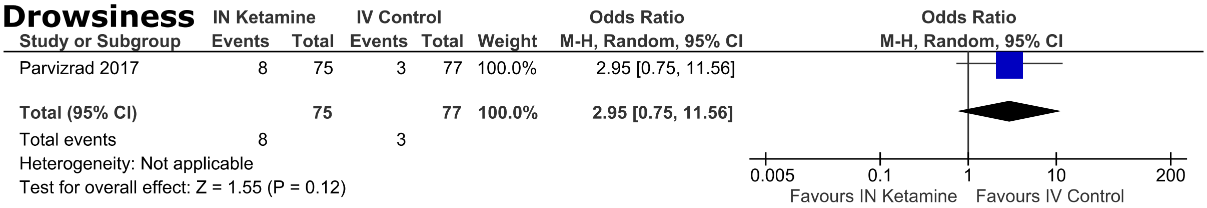

T

Euphoria

Study or Subgroup	IN Ketamine Events	IN Ketamine Total	IV Control Events	IV Control Total	Weight	Odds Ratio M-H, Random, 95% CI
Parvizrad 2017	8	75	14	77	100.0%	0.54 [0.21, 1.37]
**Total (95% CI)**		**75**		**77**	**100.0%**	**0.54 [0.21, 1.37]**
Total events	8		14			
Heterogeneity: Not applicable						
Test for overall effect: Z = 1.30 (P = 0.19)						

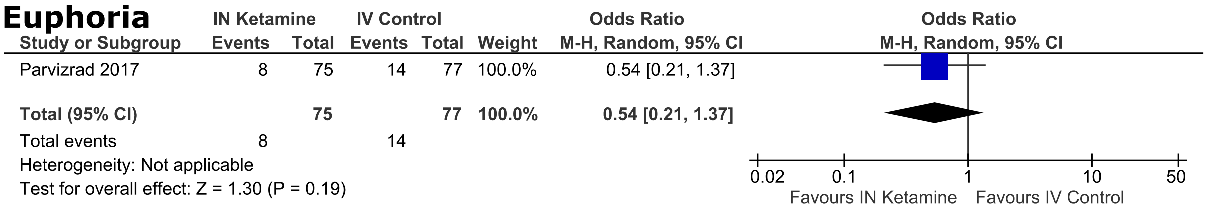

U

Headache

Study or Subgroup	IN Ketamine Events	IN Ketamine Total	IV Control Events	IV Control Total	Weight	Odds Ratio M-H, Random, 95% CI
Mozafari 2019	3	65	1	65	100.0%	3.10 [0.31, 30.58]
**Total (95% CI)**		**65**		**65**	**100.0%**	**3.10 [0.31, 30.58]**
Total events	3		1			
Heterogeneity: Not applicable						
Test for overall effect: Z = 0.97 (P = 0.33)						

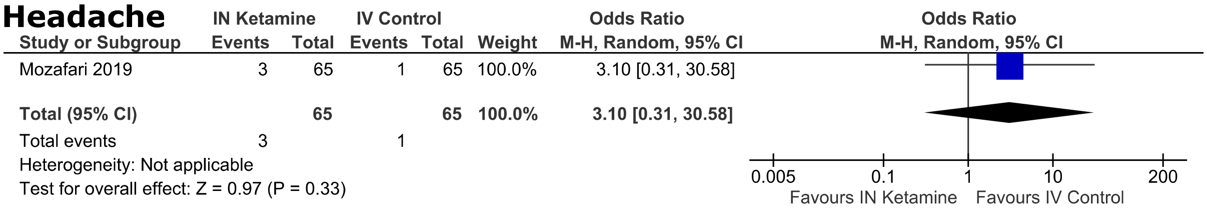

V

Hypotension

Study or Subgroup	IN Ketamine Events	IN Ketamine Total	Control Events	Control Total	Weight	Odds Ratio M-H, Random, 95% CI
Farnia 2017	0	20	8	20	100.0%	0.04 [0.00, 0.68]
**Total (95% CI)**		**20**		**20**	**100.0%**	**0.04 [0.00, 0.68]**
Total events	0		8			
Heterogeneity: Not applicable						
Test for overall effect: Z = 2.22 (P = 0.03)						

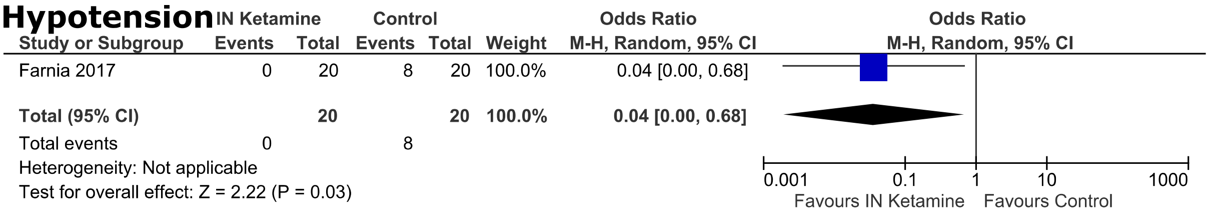

Figure S1. Prevalence (A-K) and odds ratio (L-V) of adverse events.
