# Supplementary material for: Efficacy and Safety of Intranasal Ketamine for Acute Pain Management in the Emergency Setting: A Systematic Review and Meta-Analysis
Source: J Clin Med. 2021 Sep 2;10(17):3978. doi: 10.3390/jcm10173978 (PMC8432265; doi:10.3390/jcm10173978)
Supplement: Supplementary file 1 [file jcm-10-03978-s001.zip › Table S1_Search Strategy.pdf]

Table S1. Search strategies.

Databases	Search strategies
**PubMed**	((Ketamine[Title/Abstract]) AND (pain[Title/Abstract])) AND (emergency[Title/Abstract])
**Scopus**	TITLE(Ketamine) AND TITLE-ABS-KEY(pain) AND TITLE-ABS-KEY(emergency)
**Google Scholar**	allintitle: ketamine acute pain
**Web of Science**	TI=(Ketamine) AND TI=(pain) AND TI=(emergency)
**Cochrane Database**	Record title: Ketamine AND pain AND emergency
