# Supplementary material for: Efficacy and Safety of Intranasal Ketamine for Acute Pain Management in the Emergency Setting: A Systematic Review and Meta-Analysis
Source: J Clin Med. 2021 Sep 2;10(17):3978. doi: 10.3390/jcm10173978 (PMC8432265; doi:10.3390/jcm10173978)
Supplement: Supplementary file 1 [file jcm-10-03978-s001.zip › Table S2_Quality assessment.pdf]

Table S2. Quality assessment of the included randomised controlled trials.

No.	Study ID	Questions assessing the included studies													
		**1**	**2**	**3**	**4**	**5**	**6**	**7**	**8**	**9**	**10**	**11**	**12**	**13**	**Yes (%)**
1	Bouida 2020	Y	Y	Y	Y	Y	Y	Y	Y	Y	Y	Y	Y	Y	100%
2	Farnia 2017	Y	Y	Y	Y	Y	Y	Y	Y	Y	Y	Y	Y	Y	100%
3	Forouzan 2017	Y	U	Y	U	U	U	Y	Y	Y	Y	Y	Y	Y	69%
4	Mozafari 2019	Y	Y	N	Y	Y	Y	Y	Y	Y	Y	Y	Y	Y	92%
5	Parvizrad 2017	Y	Y	Y	Y	Y	Y	Y	Y	Y	Y	Y	Y	Y	100%
6	Pouraghaei 2020	Y	Y	Y	Y	Y	Y	Y	N	Y	Y	Y	Y	Y	92%
7	Shimonovich 2016	Y	U	Y	U	U	U	Y	Y	Y	Y	Y	Y	Y	69%

1. Was true randomisation used for assignment of participants to treatment groups? 2. Was allocation to treatment groups concealed? 3. Were treatment groups similar at the baseline? 4. Were participants blind to treatment assignment? 5. Were those delivering treatment blind to treatment assignment? 6. Were outcomes assessors blind to treatment assignment? 7. Were treatment groups treated identically other than the intervention of interest? 8. Was follow up complete and if not, were differences between groups in terms of their follow up adequately described and analysed? 9. Were participants analysed in the groups to which they were randomised? 10. Were outcomes measured in the same way for treatment groups? 11. Were outcomes measured in a reliable way? 12. Was appropriate statistical analysis used? 13. Was the trial design appropriate, and any deviations from the standard RCT design (individual randomisation, parallel groups) accounted for in the conduct and analysis of the trial? Y=Yes; N=No; U=Unclear.
